# Can We Apply National Cancer Grid of India Consensus Guidelines for the Management of Cervical Cancer in Low-Resource Settings?

**DOI:** 10.1200/JGO.18.00074

**Published:** 2018-06-21

**Authors:** Linus Chuang

**Affiliations:** Western Conneticut Health Network, Larner College of Medicine at University of Vermont, Danbury, VT

Cervical cancer affects more women in low-resource than in high-resource countries. Globally, 85% of the 528,000 incident cases and 87% of the 266,000 deaths from cervical cancer occur in women from low-resource countries.^1^ In many of these countries, cervical cancer is the second most common form of cancer in women, and, strikingly, in one half of the Sub-Saharan and some Central American countries, it is the most prevalent form of cancer.^2^ High mortality is a result of multiple compounding factors that begins with a lack of screening programs; thus, most patients present with advanced-stage cancers, the treatment of which is compromised by the paucity of effective treatment modalities, including surgeons who are trained to perform radical hysterectomy, radiation machines, and chemotherapy. Both National Comprehensive Cancer Network (NCCN) and ASCO published their respective first resource-stratified clinical practice guidelines with recommendations on alternative best treatment options for clinicians who practice in these countries.^3,4^ The ASCO guideline was developed on the basis of the review of existing guidelines or expert consensus opinions when evidence was not available.^5,6^ The European Society of Medical Oncology has also recently developed a cervical cancer guideline for clinicians who practice in Europe.^7^ On the resource-stratified guidelines, a four-tiered approach—basic, limited, enhanced, and maximal—was developed on the basis of recommendations by the Breast Health Global Initiative.^8^ The ASCO guideline was written by a panel of international experts from Africa, Asia, America, and Europe, including those in medical oncology, gynecologic oncology, radiation oncology, palliative care, health economics, obstetrics, and gynecology, as well as the patient advocacy group. They evaluated existing literature and similar guidelines and reviewed cost-effective analyses to determine how best to develop the guidelines for each tier. Of note, the recommendations are intended to complement, but not replace, local guidelines. The ASCO guideline is meant to help make an impact for women who are diagnosed with cervical cancer around the world and to facilitate the ability of local governments to stay abreast of what needs to be done and at what they should be aiming in patients with cervical cancer.

In each tier on the resource-stratified guideline, and for each stage of cervical cancer, ASCO and NCCN recommendations discuss optimal therapy, which could include a combination of radiation, chemotherapy, and/or surgery, depending on the respective clinical settings and stages of the diseases, as well as palliative care and pain management. It is hoped that the guideline will serve as a tool for clinicians to show policymakers in their regions what is possible with their resources for women who are diagnosed with cervical cancer—often young women in their most productive roles in society. The guideline recommends less radical surgery, such as extrafascial hysterectomy or its modification, for patients with stage IA2, IB1, or IIA1 disease if the surgical capacity is present and the disease can be removed safely with negative margin. For more advanced-stage disease, neoadjuvant chemotherapy (NACT), followed by extrafascial hysterectomy with modification has been recommended when feasible. Two randomized phase III trials (European Organization for Research and Treatment of Cancer 55994 and ClinicalTrials.gov identifier: NCT00193739) are comparing NACT followed by surgery with primary chemoradiation for patients with stage IB2 to IIIA disease. Results of the NCT00193739 trial that was conducted in India was presented at the European Society of Medical Oncology in September 2017. It enrolled 633 patients to compare the outcomes of patients who underwent three cycles of NACT every 3 weeks followed by radical hysterectomy versus conventional chemoradiation (CCRT). Disease-free survival was 69.3% versus 76.7% in favor of CCRT; however, there were no differences in overall survival between the two regimens (ClinicalTrials.gov identifier: NCT00039338).^9^ This study may add impetus to the role of NACT in the management of cervical cancer. Significantly, in settings in which radiation therapy is not readily available, NACT followed by surgery may remain an alternative best option. For settings in which resources are available for patients to be treated in a timely fashion, CCRT is recommended for the management of locally advanced cervical cancer from stage IIB to IVA.

With the article that accompanies this work, Chopra et al^10^ have published an important paper on the National Cancer Grid of India consensus guideline for the management of cervical cancer. This is a relevant paper in the context of addressing the burden of cervical cancer in India. According to a 2014 WHO report, implementation of standard of care is an important step in the management of the disease in the country, especially considering the operational framework for the management of common cancers, which includes cervical cancer.^11^ The guideline represents a consensus statement of the gynecologic cancer expert group with the hope of assisting in the homogenization of the management of cervical cancer in India. The consensus panel addressed the framework of the questions with the PubMed Database and the Cochrane database for systematic reviews. Although there was no formal tool used to score the evidence, recommendations were made on the basis of the best available evidence for the clinical context; recommendations by the expert panels were provided when there was a lack of level I evidence.

In this Indian National Cancer Grid guideline, magnetic resonance imaging and contrast-enhanced computed tomography scan were recommended as optimal and minimal options, respectively, for imaging in patients with early and locally advanced cervical cancer. This is in agreement with recommendations in the ASCO and NCCN guidelines on the work-up for high-resource settings. Positron emission tomography scan was not recommended as it does not have specificity over computed tomography scan in predicting nodal metastasis. The guideline provides templates for magnetic resonance imaging and histopathology reports with the intention of maintaining a high standard for clinical practice. Completion of 8 weeks of radiation was emphasized, which is vital as survival outcomes are associated with prolonged radiation therapy for patients with cervical cancers. Furthermore, data from Indian trials were included to support the recommendations in the guideline. For example, intensity-modulated radiation therapy was compared with three-dimensional conformal radiation. Although the outcome was in favor of intensity-modulated radiation therapy, as it is associated with a reduced incidence of late bowel toxicity, the difference was not statistically significant.^12,13^ A second trial—the aforementioned NCT00193739 trial—compared NACT followed by surgery with concurrent CCRT recently reported by Gupta et al. On the basis of the finding of superior 5-year disease-free survival, although overall survival was similar, CCRT remains the standard treatment for patients with locally advanced cervical cancer.^9^ Absent is the discussion of the management of patients with cervical cancer when radiation therapy is not readily accessible. Should NACT be considered as an acceptable alternative? A critical question raised was whether the recommended guideline for cervical cancer radiation in India can be adequately implemented given the relative shortage of radiation machines and accessibility for financially deprived patients. The authors state that, because less than one half of patients received CCRT in India, the judicious use of chemotherapy during radiation therapy should be encouraged to improve survival outcomes. However, it should be noted that radiation therapy should not be delayed if chemotherapy is not readily available.^14^

The number of new cancer cases in India has been rising rapidly. In 2008, the number was 0.95 million, but is projected to increase to 1.7 million by 2035. Although the incidence of cancer in India is lower than that in Europe and North America, the mortality rate is higher, which suggests low effectiveness in health care.^15^ In the WHO country cancer profiles published in 2014, there were 0.4 high-energy teletherapy units per million inhabitants, with a total of 353 radiation oncologists working in 314 radiotherapy centers in India.^16^ Both radiotherapy and chemotherapy are mostly available, whereas oral morphine is not generally available in the public health system. This is in contrast to five high-energy teletherapy units per million inhabitants, with 161 radiation oncologists working in 69 radiotherapy units in the United Kingdom.^17^ The annual incidence of cervical cancer in 2012 was 122,844 with an age-standardized rate of 22 in India, whereas it was 2,659 with an age-standardized rate of 7.1 in the United Kingdom. This would translate into ten-fold more patients treated at each center in India than in the United Kingdom; therefore, the burden of disease is significantly higher and the centers that can offer treatment are fewer in India than in any other high-resource country. One of India’s major public health challenges has been affordable and equitable cancer care infrastructures. The guideline proposed by the authors is notable as it sets the standard for the best care that can be provided to women with cervical cancer in India.^18^ There remain challenges in linking the recommendations and guidelines to the reality of Indian cervical cancer care; reality is reflected in the limited number of centers that provide radiation therapy to address the high burden of disease. The authors hold that CCRT remains the standard of care in India and perhaps in all settings where radiation machines are available. One may consider NACT followed by surgery as acceptable in selected patients in settings where radiation therapy is still not readily available in India or in other regions of the world, such as Africa. The authors relate that efforts are being made to audit and report compliance within the Indian institutions that participated in the development of this guideline. This is an excellent approach and much emphasis is needed on improving treatment capacity, including increases in the numbers of radiation machines and radiation oncologists. This guideline will serve as a wake-up call for policymakers in India to increase radiation capacity, at least doubling what is available today to reach a minimum of one teletherapy unit per million inhabitants.^19^ Ultimately, improving the survival rate will depend on diagnosing cervical cancer during the earlier stages of the disease. Downstaging of the disease or reducing the incidence of cervical cancer will rely on human papillomavirus vaccination and cervical cancer screening, which are crucial for improving survival.^20^
